# Psychological Inflexibility and Psychological Distress in Adults: The Mediating Role of Resilience

**DOI:** 10.3390/bs16091632

**Published:** 2026-09-13

**Authors:** Miluska Moreyra-Ruiz, Jonatan Baños-Chaparro

**Affiliations:** 1 Escuela de Posgrado, Universidad Ricardo Palma, Lima 15046, Peru; 202321112@urp.edu.pe; 2 Programa Académico de Psicología, Facultad de Ciencias de la Salud, Universidad Privada Norbert Wiener, Lima 15046, Peru

**Keywords:** psychological inflexibility, resilience, psychological distress, adults, mental health

## Abstract

**Introduction**: The high prevalence of anxiety and depression in adults affects their overall functioning. Within Relational Frame Theory, psychological inflexibility is theoretically associated with avoidance of private events and has been linked to greater anxiety and depressive symptoms and lower resilience. However, the integration of these constructs into explanatory models remains limited, restricting the understanding of their interrelationships and potentially relevant protective resources. **Objective**: To examine the indirect association between psychological inflexibility and psychological distress through resilience within a theoretically specified cross-sectional model. **Methods**: A cross-sectional design with latent variables was employed using a quantitative approach. The sample consisted of 950 Peruvian adults (64% women), recruited in Metropolitan Lima, who completed a sociodemographic questionnaire and psychological measures. Statistical analyses were conducted using structural equation modeling. **Results**: The proposed model demonstrated adequate fit (robust CFI = 0.94, robust RMSEA = 0.05, 90% CI [0.050, 0.057], SRMR = 0.04). Psychological inflexibility showed a strong positive direct association with psychological distress (β = 0.803, *p* < 0.001) and a significant indirect association through resilience (β = 0.073, *p* < 0.001]). This pattern was consistent with complementary mediation within the specified cross-sectional model. **Conclusions**: Higher psychological inflexibility was associated with lower resilience and greater psychological distress. Resilience statistically accounted for a small but significant portion of the association between psychological inflexibility and distress, whereas the direct association remained predominant. Given the cross-sectional design, these findings should not be interpreted as evidence of temporal or causal relationships. Longitudinal research is required to examine the directionality of these associations.

## 1. Introduction

Mental disorders affect one in seven people worldwide; in particular, depression and anxiety are the most prevalent mental health conditions (World Health Organization, 2022). This distress negatively impacts quality of life and impairs individuals’ functional capacity (García-Sancho et al., 2018). Adults represent the most affected group: according to the 2021 global report, 287 million adults experienced anxiety out of a total of 359 million cases, and 275 million adults suffered from depression out of 280 million cases in 2019 (World Health Organization, 2022). Similarly, in Peru, treated cases in the adult population show a high concentration of anxiety and depressive disorders; a higher prevalence has also been identified in urban areas, particularly in Metropolitan Lima (Saavedra et al., 2023). Indeed, this distress has broader social implications, as it significantly affects families and communities by increasing healthcare demands and reducing productivity.

From a broader mental health perspective, anxiety and depressive symptoms frequently coexist and can be understood as components of psychological distress, a construct that captures nonspecific emotional suffering rather than a single psychiatric condition (Ali et al., 2023). This perspective is particularly relevant because psychological distress may be present across different levels of symptom severity and may interfere with emotional, interpersonal, occupational, and daily functioning even when diagnostic criteria for a specific mental disorder are not met. Consequently, identifying psychological processes that contribute to the emergence or maintenance of distress is important not only for explaining psychopathology but also for developing preventive and transdiagnostic interventions. In this regard, approaches focused exclusively on symptom categories may provide an incomplete explanation if the underlying psychological processes shared by anxiety and depression are not considered.

Within this transdiagnostic perspective, psychological inflexibility has been identified as a relevant psychological process associated with psychological distress (Avsec et al., 2022; Mohajeri et al., 2023). Meta-analytic evidence has also shown consistent associations between psychological inflexibility and different mental health problems, supporting its relevance as a transdiagnostic process rather than as a factor restricted to a particular disorder (Yao et al., 2023). Thus, examining psychological inflexibility may contribute to understanding why individuals exposed to different stressors develop or maintain symptoms of anxiety and depression.

### 1.1. Theoretical Framework

#### 1.1.1. Psychological Inflexibility and Psychological Distress

The proposed model is grounded first in Relational Frame Theory (RFT) and the psychological flexibility model underlying Acceptance and Commitment Therapy (ACT). RFT provides a functional account of human language and cognition in which verbally established relations can alter the psychological functions of internal and external events. ACT extends this account to a model of psychological functioning in which psychological inflexibility emerges when behavior becomes excessively governed by verbal content and by attempts to avoid, suppress, or control unwanted private experiences (Hayes et al., 2006; Bond et al., 2011).

From this perspective, psychological inflexibility does not refer simply to experiencing unpleasant thoughts or emotions. Rather, it involves rigid responding to these experiences in ways that reduce sensitivity to contextual demands and restrict behavior that is consistent with personally relevant goals or values. Experiential avoidance may provide short-term relief; however, when avoidance becomes a dominant regulatory strategy, the behavioral repertoire available for responding to difficult situations becomes increasingly restricted. Consequently, aversive internal experiences may acquire greater influence over behavior and contribute to the persistence of anxiety, depressive symptoms, and other forms of psychological distress (Hayes et al., 2006; Kashdan & Rottenberg, 2010).

Empirical findings are consistent with this theoretical proposition. Psychological inflexibility has been associated with poorer psychological functioning and with symptoms of anxiety and depression, while adaptive coping processes may partly explain these relationships (Avsec et al., 2022; Mohajeri et al., 2023). This suggests that the association between psychological inflexibility and distress may involve not only a direct pathway but also indirect mechanisms related to individuals’ psychological resources for responding to adversity. Identifying these mechanisms is important because they may represent modifiable protective factors that could be strengthened through psychological intervention.

This theoretical account provides the basis for expecting a direct relationship between psychological inflexibility and psychological distress. Accordingly, the first hypothesis was formulated:

**H1.**  *Psychological inflexibility will be positively associated with psychological distress.*

#### 1.1.2. Psychological Inflexibility and Resilience

The second component of the theoretical framework concerns resilience. Resilience has been conceptualized in different ways across the psychological literature, including as a capacity, process, or pattern of adaptive functioning in the context of adversity (Southwick et al., 2014). Consistent with the conceptual foundation of the Connor–Davidson Resilience Scale, the present study approaches resilience primarily as an adaptive psychological capacity related to coping with stress and recovering or maintaining functioning when facing difficult circumstances (Connor & Davidson, 2003). Because the present study is cross-sectional and relies on self-report measures, resilience is therefore operationalized as perceived adaptive capacity rather than as an objectively demonstrated longitudinal trajectory following a specific adverse event.

Psychological inflexibility and resilience are conceptually related but distinct. Psychological inflexibility describes a regulatory process characterized by rigid responding to private experiences, whereas resilience refers more broadly to the capacity to mobilize adaptive resources when facing stress or adversity. The psychological flexibility literature emphasizes that adaptive functioning requires the ability to modify behavioral repertoires according to changing situational demands rather than persist with ineffective responses (Kashdan & Rottenberg, 2010). From this perspective, persistent experiential avoidance and rigid behavioral regulation may interfere with adaptation because they reduce the range of responses available to deal with stressful circumstances. Individuals whose behavior is strongly organized around avoiding unwanted thoughts or emotions may therefore have greater difficulty tolerating distress, adjusting coping responses, and mobilizing psychological resources when adversity occurs.

Empirical evidence is consistent with this theoretical reasoning. Psychological inflexibility has been negatively associated with resilience (Buyukoksuz & Tekin, 2023), whereas higher resilience has been associated with lower anxiety and depressive symptoms across different stressful contexts (Avsec et al., 2022).

This theoretical reasoning suggests that greater psychological inflexibility should be associated with lower resilience. It also suggests that resilience should be inversely associated with psychological distress because greater adaptive capacity provides individuals with more resources to maintain or recover functioning under stressful circumstances. These arguments lead to the following hypotheses:

**H2.**  *Psychological inflexibility will be negatively associated with resilience.*

**H3.**  *Resilience will be negatively associated with psychological distress.*

#### 1.1.3. The Mediating Role of Resilience

Integrating these two perspectives provides the theoretical rationale for the proposed mediation model. The ACT model predicts a direct association between psychological inflexibility and psychological distress because rigid attempts to regulate or avoid private experiences can themselves maintain emotional suffering. At the same time, the adaptive-capacity perspective on resilience suggests a second pathway. Psychological inflexibility may be associated with a more restricted capacity to respond adaptively to stressful circumstances; reduced resilience may subsequently be associated with greater psychological distress because fewer psychological resources are available to cope with adversity.

Resilience is therefore not conceptualized as a component of psychological flexibility or simply as the opposite of psychological inflexibility. Instead, it represents a theoretically distinct adaptive capacity through which part of the association between psychological inflexibility and psychological distress may be statistically accounted for. This distinction is important because it allows direct and indirect pathways to coexist within the proposed model. Psychological inflexibility may remain strongly associated with psychological distress while also showing an indirect association through lower resilience.

The plausibility of an indirect pathway is also supported by evidence showing that associations between psychological inflexibility and psychological outcomes may involve intermediate adaptive processes, including coping, self-compassion, mindfulness, and emotion regulation (Avsec et al., 2022; Buyukoksuz & Tekin, 2023; Buyukoksuz & Kayaal-Pehlivan, 2025). These findings suggest that psychological inflexibility may be associated with distress not only directly but also through its relationship with adaptive psychological resources.

Although previous research supports the individual associations among these constructs, it remains unclear whether resilience statistically accounts for a meaningful portion of the association between psychological inflexibility and psychological distress. The contribution of the proposed model therefore lies in testing resilience as a distinct adaptive pathway while simultaneously estimating the direct association between psychological inflexibility and distress.

On this basis, the final hypothesis was proposed:

**H4.**  *Resilience will statistically mediate the association between psychological inflexibility and psychological distress, such that greater psychological inflexibility will be associated with lower resilience, which in turn will be associated with greater psychological distress.*

Given the cross-sectional nature of the study, these hypotheses represent theoretically ordered associations and should not be interpreted as predictions of temporal or causal relationships.

### 1.2. The Present Study

Based on a review of the available literature, few studies were found that examine the relationship among these three constructs within a structural model in the Peruvian adult population. This lack of evidence is particularly important in the Peruvian context, where anxiety and depressive problems represent an important mental health concern and a substantial concentration of treated cases has been reported in Metropolitan Lima (Saavedra et al., 2023). Evidence obtained in other cultural contexts cannot automatically establish that the same explanatory relationships operate with comparable strength among Peruvian adults. Examining these constructs within this population may therefore contribute both to the international literature on transdiagnostic psychological processes and to the development of locally relevant approaches to mental health assessment and intervention.

This gap limits understanding of how maladaptive regulatory processes and adaptive psychological resources may operate jointly in relation to psychological distress. From an applied perspective, examining resilience as a potential intermediate mechanism linking psychological inflexibility with distress could also have implications for intervention. Psychological treatments could potentially address both maladaptive processes, such as experiential avoidance and psychological rigidity, and protective resources associated with adaptive coping. Such an approach would be compatible with the broader need for psychological interventions that target common mechanisms underlying anxiety and depressive symptoms rather than addressing these manifestations exclusively as separate problems (García-Sancho et al., 2018).

## 2. Materials and Methods

### 2.1. Design

The study was conducted using an associative strategy with an explanatory design based on a mediation model including both latent and observed variables (Ato et al., 2013). It also followed a quantitative, basic, and cross-sectional approach.

### 2.2. Participants

A total of 950 adults from Metropolitan Lima, Peru, participated in the study. Participants were selected through non-probabilistic convenience sampling and met the following inclusion criteria: (a) aged between 18 and 59 years, (b) literate adults, and (c) Peruvian nationality and residence in Metropolitan Lima. Exclusion criteria were: (a) diagnosis of an intellectual or neurological developmental disorder that would prevent completion of the survey, (b) incomplete responses in the sociodemographic section or psychological instruments, and (c) voluntary withdrawal from the survey.

For sample size estimation, the Soper calculator (version 4.0), appropriate for structural equation modeling, was used. Based on a structural model with three latent variables and 27 observed variables, an anticipated effect size of 0.20 was specified as a conservative minimum effect of substantive interest, allowing the study to be adequately powered to detect small-to-moderate associations among the latent variables (Westland, 2010; Soper, 2025). Assuming a statistical power of 0.80 and a significance level of 0.05, the minimum recommended sample size to detect the specified effect was 296. The final sample exceeded this recommendation.

Regarding participant characteristics, most were women (64%), with a mean age of 23.4 years (SD = 7.6; range: 18–59 years). At the time of the survey, 51.2% were only studying, 15.2% were only working, and 33.7% were both studying and working. In terms of residence, most participants lived in North Lima (54.2%), followed by South Lima (27.7%), Central Lima (9.3%), Callao (5.4%), and East Lima (3.5%).

### 2.3. Instruments

Kessler Psychological Distress Scale (K10). The K10 consists of 10 items measuring nonspecific symptoms of anxiety and depression over the past four weeks. Responses are recorded on a five-point Likert scale. Total scores range from 10 to 50, with higher scores indicating greater psychological distress. In this study, the Spanish-language version evaluated in Peruvian university students from Arequipa (Arias-Gallegos et al., 2019) was used. This study reported evidence regarding the factorial structure and reliability of the K10 in that population. In the present sample, good internal consistency was observed (ω = 0.91).

Acceptance and Action Questionnaire-II (AAQ-II). This questionnaire consists of 7 items measuring psychological inflexibility. Each item is rated on a 7-point scale. Total scores range from 7 to 49, with higher scores reflecting greater psychological inflexibility. The Spanish-language version, evaluated in Peruvian university students from Lima (Valencia & Falcón, 2022), was used. The study reported psychometric evidence of validity and reliability for the AAQ-II in that population. In the present sample, favorable reliability was confirmed (ω = 0.91).

Connor–Davidson Resilience Scale—10 items (CD-RISC-10). This scale consists of 10 items measuring resilience. Responses are recorded on a five-point Likert scale ranging from 0 (never) to 4 (almost always). Total scores range from 0 to 40, with higher scores indicating greater resilience. In this study, the Spanish-language version, evaluated in Peruvian university students from Metropolitan Lima (Bernaola Ugarte et al., 2022), was administered. The study reported psychometric evidence of validity and reliability for the CD-RISC-10 in that population. Good reliability was observed in the present sample (ω = 0.87).

### 2.4. Statistical Analysis

Statistical processing was conducted using the open-source software RStudio 4.5.2. In the first phase, descriptive analyses of the psychological variables were performed, including measures of central tendency and dispersion such as the mean and standard deviation. Associations between variables were examined using Pearson’s correlation coefficient, with effect sizes interpreted as small (r = 0.10), medium (r = 0.20), large (r = 0.30), and very large (r = 0.40) (Funder & Ozer, 2019).

In the second phase, the measurement model was evaluated through confirmatory factor analysis (CFA) using the 27 item-level indicators corresponding to resilience, psychological inflexibility, and psychological distress. Given the significant departure from multivariate normality, the measurement model was estimated using robust maximum likelihood (MLR) (Muthén & Muthén, 2017). Model fit was evaluated using the scaled chi-square statistic (χ^2^), robust Comparative Fit Index (CFI > 0.90), robust Tucker–Lewis Index (TLI > 0.90), robust Root Mean Square Error of Approximation (RMSEA < 0.08), and Standardized Root Mean Square Residual (SRMR < 0.08) (Jordan, 2021). Standardized factor loadings and latent-factor correlations were examined. Discriminant validity was evaluated by examining latent-factor correlations and complementary CFA-based procedures, including constrained-correlation tests and comparisons with alternative models in which pairs of latent factors were merged (Rönkkö & Cho, 2022). HTMT2 coefficients were also calculated as an additional descriptive indicator of construct overlap (Henseler et al., 2015). Given the high association between psychological inflexibility and psychological distress, particular attention was paid to the empirical distinction between these two constructs.

In the third phase, a cross-sectional mediation model was tested using structural equation modeling (SEM). Given the significant departure from multivariate normality, the structural model was also estimated using MLR, which provides robust standard errors and scaled test statistics that are less sensitive to violations of multivariate normality. The observed indicators were treated as approximately continuous because they contained five or more response categories and the sample size was large (N = 950) (Rhemtulla et al., 2012). No missing data were present in the final analytical dataset; therefore, no missing-data treatment or imputation procedure was required. Model fit was evaluated using the scaled chi-square statistic, robust CFI, robust TLI, robust RMSEA with its 90% confidence interval, and SRMR. At the local level, standardized regression coefficients (β) and effect sizes were evaluated and interpreted as minimal (β = 0.10), moderate (β = 0.50), and strong (β = 0.80) (Ferguson, 2016). A statistical significance level of *p* < 0.05 was used. Mediation was evaluated based on the estimated indirect association and its 95% confidence interval. When the indirect association was statistically significant and the direct association remained significant in the same direction, the pattern was interpreted as complementary mediation.

### 2.5. Procedure

Data collection was conducted virtually through an online questionnaire administered between September and October 2025. The survey was developed and hosted on the Google Forms platform and included the informed consent form. It was distributed through the researchers’ social media networks (Facebook, LinkedIn, and WhatsApp), which significantly expanded its reach to diverse profiles of potential participants.

Before participating, respondents received comprehensive information about the study objectives, the anonymous nature of their participation, the strictly academic use of the collected data, and the procedures related to data handling. Informed consent was also required as an essential condition for proceeding with the questionnaire. At this stage, four participants were excluded because they did not meet the inclusion criterion of residing in Metropolitan Lima.

The use of surveys in digital environments offers several methodological advantages. These include broader access to the target population, the possibility of systematically managing and monitoring responses, flexibility in the use of different dissemination channels, and greater efficiency in terms of time and costs during the data collection process. These characteristics contribute to optimizing both the available resources and the quality of the research process (Gosling & Mason, 2015).

### 2.6. Ethical Considerations

The study was conducted in accordance with the ethical guidelines of the American Psychological Association (APA) and the Code of Ethics of the Colegio de Psicólogos del Perú (CPsP), as outlined in Chapter Six on good research practices (CPsP, 2024; Knapp & VandeCreek, 2003). All participants provided informed consent prior to participation. The survey was anonymous and voluntary, and data confidentiality was ensured. The study was reviewed and approved by the Ethics Committee of the Universidad Ricardo Palma under registration number 145-2025-EPG-D.

## 3. Results

### 3.1. Descriptive Analysis

Descriptive statistics indicated that resilience had the highest mean (M = 27.13, SD = 6.61), followed by psychological distress (M = 26.57, SD = 7.92) and psychological inflexibility (M = 25.18, SD = 10.07). Regarding correlations, resilience was negatively and significantly associated with both psychological inflexibility (r = −0.49, *p* = 0.001) and psychological distress (r = −0.50, *p* = 0.001). In turn, psychological inflexibility showed a positive and significant correlation with psychological distress (r = 0.80, *p* = 0.001). Given the high observed correlation between psychological inflexibility and psychological distress, their empirical distinctiveness was further examined at the latent-variable level through the measurement model and specific discriminant-validity analyses. All effect sizes were very large.

### 3.2. Measurement Model and Discriminant Validity

**Figure 1:**
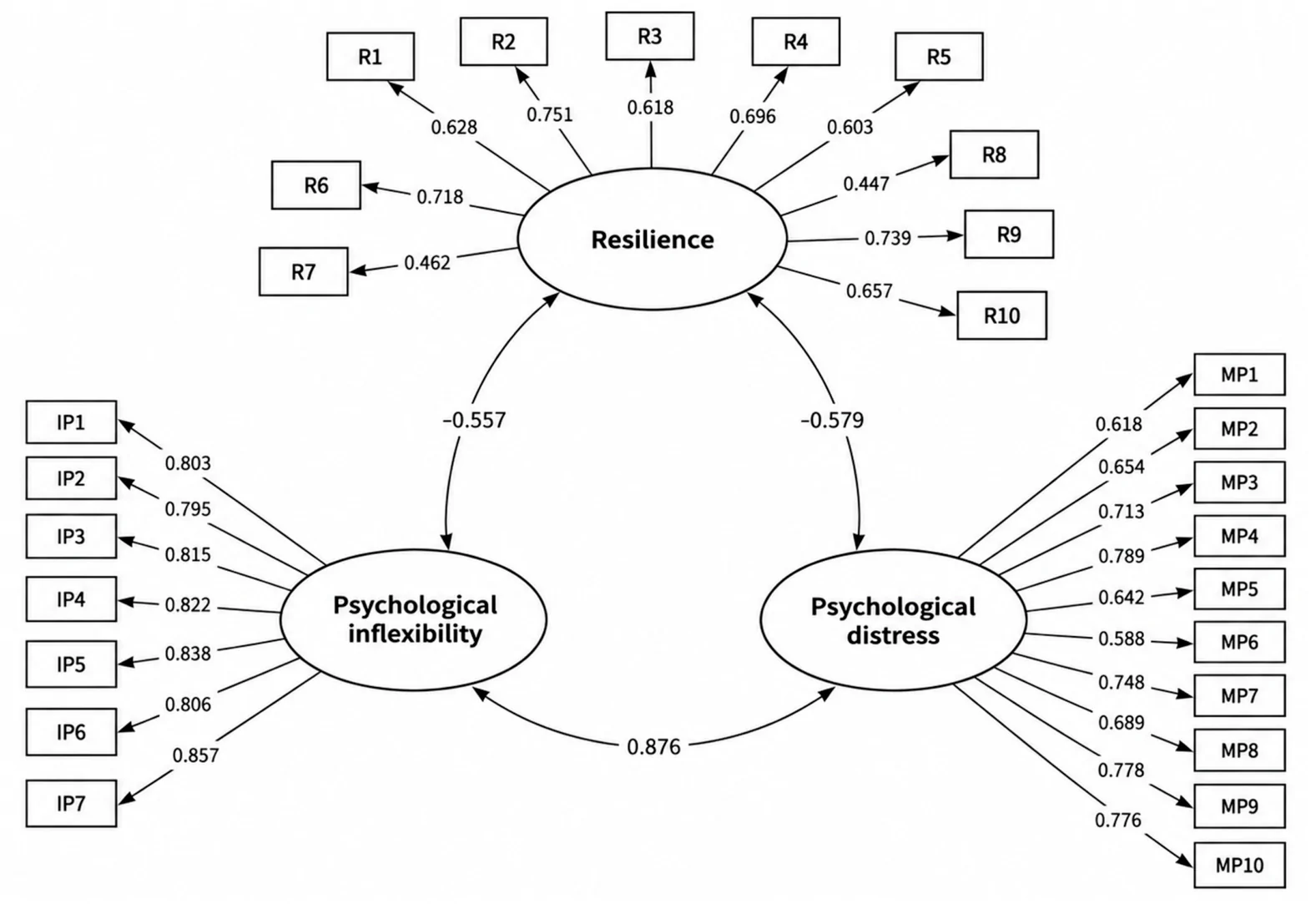
Standardized measurement model of resilience, psychological inflexibility, and psychological distress.

Before evaluating the structural model, a three-factor confirmatory factor analysis was conducted to examine the measurement model. The three-factor measurement model showed adequate robust fit to the data: scaled χ^2^(321) = 1025.69, *p* = 0.001, robust CFI = 0.939, robust TLI = 0.934, robust RMSEA = 0.054, 90% CI [0.050, 0.057], and SRMR = 0.038. Standardized factor loadings ranged from 0.447 to 0.751 for resilience, from 0.795 to 0.857 for psychological inflexibility, and from 0.588 to 0.789 for psychological distress (Figure 1).

The latent-factor correlations were −0.557 between resilience and psychological inflexibility, −0.579 between resilience and psychological distress, and 0.876 between psychological inflexibility and psychological distress. Because the latter association was high, additional discriminant-validity analyses were conducted. HTMT2 values were 0.536 for resilience–psychological inflexibility, 0.550 for resilience–psychological distress, and 0.870 for psychological inflexibility–psychological distress. Although the latter value remained below the 0.90 criterion, it indicated substantial construct overlap.

For psychological inflexibility and psychological distress, the 95% confidence interval for the latent correlation was [0.852, 0.901]. Constraining this correlation to 0.90 did not significantly worsen model fit, Δχ^2^(1) = 2.14, *p* = 0.144. However, an alternative model in which psychological inflexibility and psychological distress were merged into a single factor showed substantially poorer fit than the three-factor model, Δχ^2^(2) = 212.69, *p* < 0.001. Taken together, these findings indicate substantial overlap between psychological inflexibility and psychological distress, while the markedly poorer fit of the merged-factor model supports retaining them as empirically distinguishable constructs in the present sample.

### 3.3. Cross-Sectional Mediation Analysis

**Figure 2:**
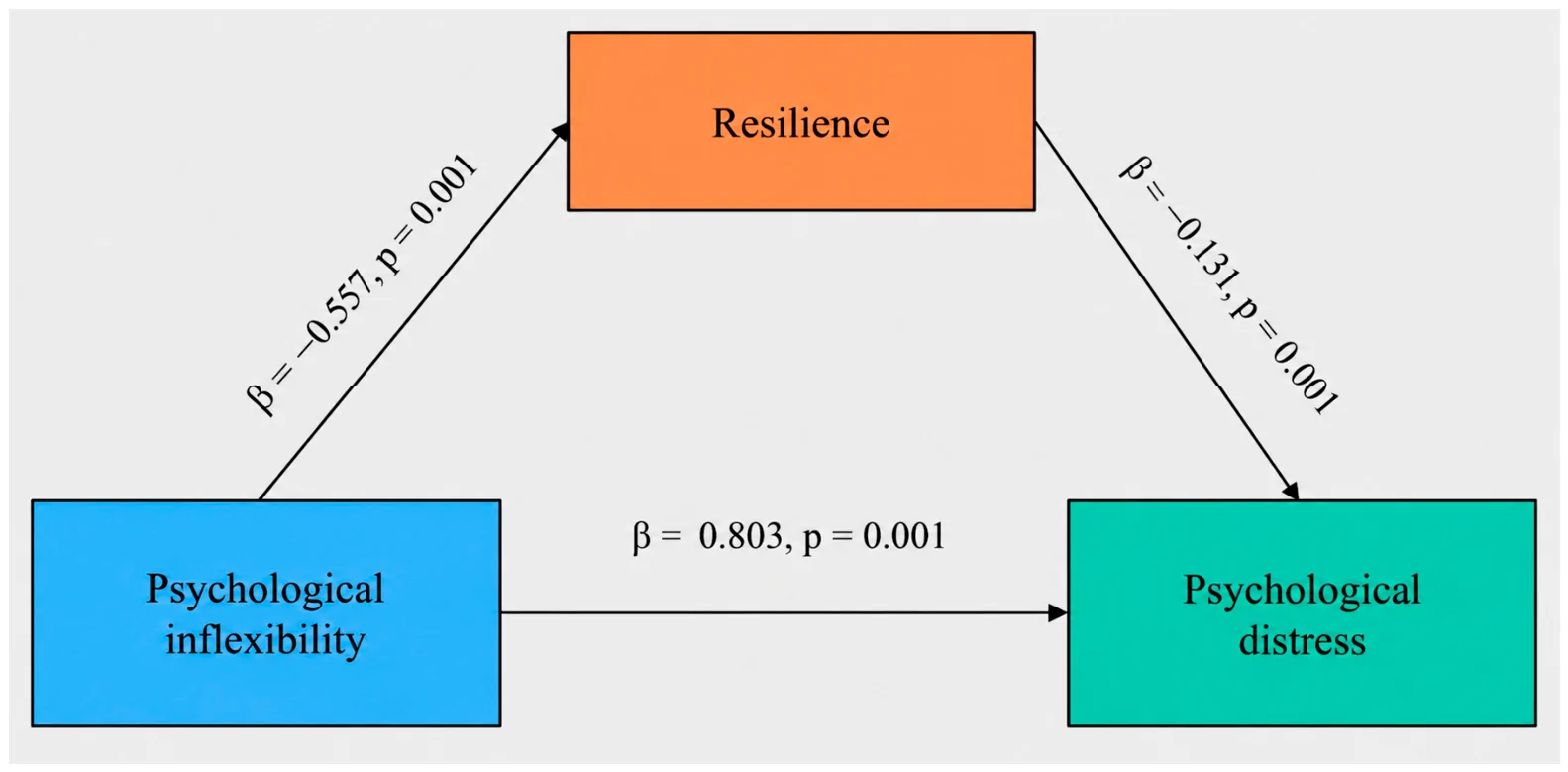
Cross-sectional mediation model of resilience in the association between psychological inflexibility and psychological distress.

The mediation model showed adequate robust fit: scaled χ^2^(321) = 1025.69, *p* = 0.001, robust CFI = 0.939, robust TLI = 0.934, robust RMSEA = 0.054, 90% CI [0.050, 0.057], and SRMR = 0.038. Results indicated that psychological inflexibility was negatively associated with resilience (β = −0.557, *p* = 0.001) and positively associated with psychological distress (β = 0.803, *p* = 0.001). In turn, resilience was negatively associated with psychological distress (β = −0.131, *p* = 0.001). The indirect association of psychological inflexibility on psychological distress through resilience was statistically significant (β = 0.073, *p* = 0.001), representing 8.3% of the total association (β = 0.876, *p* = 0.001). Since the indirect association was statistically significant while the direct association remained significant in the same direction, the observed pattern was consistent with complementary mediation within the specified cross-sectional model (see Table 1 and Figure 2).

## 4. Discussion

Psychological inflexibility has been identified as a transdiagnostic risk factor for emotional distress (Yao et al., 2023); however, its integration with resilience within an explanatory model remains empirically limited, particularly in adult populations from Latin American contexts. Within this framework, the present study aimed to examine the indirect association between psychological inflexibility and psychological distress through resilience within a cross-sectional mediation model. Importantly, the findings indicate that resilience statistically accounts for only part of this relationship, whereas the direct association between psychological inflexibility and distress remains predominant.

The mediation analysis results showed the predominance of the direct association of psychological inflexibility with psychological distress. Indeed, the association accounted for most of the total association, suggesting that psychological inflexibility constitutes a particularly relevant process associated with emotional distress, beyond the contribution of resilience. This is consistent with previous studies linking psychological inflexibility to various psychological problems such as anxiety and depression (Avsec et al., 2022; Mohajeri et al., 2023). It is also consistent with meta-analytic evidence indicating that psychological inflexibility is associated with a broad range of mental health problems, reinforcing its characterization as a transdiagnostic process (Yao et al., 2023). From the RFT perspective, psychological inflexibility may contribute to distress through verbal relations of negative affect that intensify emotional suffering. This process is central to the model of ACT, as psychological inflexibility involves a rigid dominance aimed at avoiding aversive internal experiences through experiential control strategies, which in turn contributes to the development, maintenance, and exacerbation of psychological distress (Bond et al., 2011). In fact, inflexible adults persistently seek positive private events and avoid negative ones in order to feel well at all times; however, it is inevitable that the mind generates aversive thoughts, memories, or emotions. Resisting these experiences may increase distress and reduce the capacity for a meaningful life in adults (Luciano-Soriano & Valdivia-Salas, 2006). Thus, the strong direct association observed in the present study may reflect the central role of experiential avoidance and rigid behavioral responding in the persistence of psychological distress.

Indeed, psychological inflexibility and distress do not operate solely in a direct association but also show a significant indirect association through resilience. These findings are consistent with previous research showing that coping strategies accounted for part of the association between psychological inflexibility and distress (Avsec et al., 2022). Adults with lower psychological inflexibility tend to show higher resilience scores, which were in turn associated with lower distress. The negative association identified between psychological inflexibility and resilience is also consistent with previous evidence suggesting that greater inflexibility is related to lower resilience (Buyukoksuz & Tekin, 2023). This finding suggests that rigid attempts to avoid or control internal experiences may coexist with fewer adaptive resources for responding effectively to adversity. In this context, resilience may be understood as an adaptive psychological resource associated with more favorable responses to adversity, although the present findings do not establish that resilience reduces or counteracts the influence of other psychological processes such as experiential avoidance, cognitive fusion, and disconnection from values (Bond et al., 2011). The relatively modest indirect association observed in the present study indicates that resilience statistically accounts for a smaller portion of the association between psychological inflexibility and distress, compared with the direct association. A recent study also reported indirect associations involving other intermediate variables, such as mindfulness, self-compassion, and emotion regulation (Buyukoksuz & Kayaal-Pehlivan, 2025). Therefore, this study moves away from simple unidimensional models and toward a more complex and realistic understanding of psychological variables. Future explanatory models could consequently examine resilience together with these additional processes to determine whether they operate simultaneously or sequentially in their relationship with psychological distress.

Regarding theoretical implications, the findings extend the understanding of the ACT model by integrating resilience as a statistically significant intermediate variable. This suggests that psychological inflexibility in adults is not only directly associated with symptoms of anxiety and depression but also may be related to reduced adaptive resources for coping with adversity, although other psychological processes are also involved. Conceptually, the study contributes to distinguishing resilience from psychological inflexibility: while both capture adaptive elements, resilience represents a general coping resource, whereas psychological inflexibility emphasizes rigid responses to internal experiences that interfere with behavior aligned with personal values. This distinction is relevant because resilience and psychological flexibility should not be considered interchangeable constructs; rather, they may represent complementary processes involved in adaptation to stressful experiences. At a practical level, the observed associations suggest that the joint assessment of psychological inflexibility, resilience, and psychological distress could be explored in future research as a potential approach for identifying individuals who may benefit from preventive mental-health strategies. Likewise, longitudinal and experimental studies are needed to determine whether interventions aimed at reducing psychological inflexibility and strengthening resilience can produce subsequent improvements in psychological distress. Beyond psychotherapeutic approaches, structured physical activities such as martial arts and combat sports have also been investigated in relation to mental-health outcomes in adults; however, systematic-review evidence remains heterogeneous and predominantly observational, highlighting the need for stronger longitudinal and experimental research (Ciaccioni et al., 2024).

Among the strengths of the study are the statistical robustness achieved through SEM, the large sample size, and the consistency of findings with the ACT theoretical model. The use of latent-variable modeling also allowed the hypothesized direct and indirect relationships to be examined simultaneously, providing a more comprehensive assessment than isolated bivariate associations. However, an important limitation concerns the use of mediation analysis with cross-sectional data. Although the proposed model was theoretically specified, the simultaneous measurement of psychological inflexibility, resilience, and psychological distress does not allow their temporal ordering to be established. Cross-sectional estimates of indirect effects may not accurately represent mediation processes that unfold over time and may provide biased estimates of longitudinal mediation parameters (Maxwell & Cole, 2007; O’Laughlin et al., 2018). Accordingly, the indirect association identified in the present study should be interpreted as a statistically significant indirect effect within a theoretically specified cross-sectional model, rather than as evidence that psychological inflexibility temporally precedes changes in resilience or that resilience subsequently causes changes in psychological distress. Alternative directional relationships cannot be ruled out. Longitudinal studies with repeated measurements are therefore needed to examine temporal precedence and determine whether changes in psychological inflexibility predict subsequent changes in resilience and, in turn, later psychological distress. Although the measurement-model analyses supported retaining psychological inflexibility and psychological distress as separate latent constructs, their high latent correlation (r = 0.876), HTMT2 value (0.870), and confidence interval extending slightly above 0.90 indicate substantial overlap. Therefore, associations involving these constructs should be interpreted with caution, and future studies should examine whether their empirical distinction is replicated using alternative measures and independent samples. Additionally, the use of convenience sampling resulted in a predominantly young and education-engaged sample. Participants had a mean age of 23.4 years, and most were either studying exclusively (51.2%) or simultaneously studying and working (33.7%). The sample also included a higher proportion of women (64%). This composition limits the generalizability of the findings to older adults, individuals who are not engaged in formal education, and populations with different sociodemographic characteristics, including adults from regions of Peru outside Metropolitan Lima. Future studies should therefore recruit more age-diverse and socioeducationally heterogeneous samples, preferably using probabilistic sampling when feasible. The exclusive use of self-report measures and the absence of other psychological variables (e.g., psychological flexibility, cognitive rumination, self-compassion, mindfulness, and emotion regulation) that may operate as parallel intermediate variables should also be considered. Future research could incorporate longitudinal designs and multiple intermediate variables to examine the relative contribution of resilience compared with other adaptive and maladaptive psychological processes.

## 5. Conclusions

This study shows that psychological inflexibility is not only directly associated with affective symptomatology, but that a significant indirect association with psychological distress was observed through resilience. The findings suggest that psychological inflexibility is strongly associated with psychological distress, while resilience represents an additional, although comparatively smaller, pathway through which these variables are related. Therefore, the findings suggest that resilience may be considered one of several adaptive psychological resources associated with lower psychological distress, rather than a causal mechanism linking psychological inflexibility and distress. These findings provide a basis for future longitudinal and experimental research examining whether interventions targeting psychological inflexibility and adaptive coping resources are associated with subsequent changes in psychological distress. However, given the cross-sectional design, these findings do not establish temporal or causal mediation, and alternative directional relationships cannot be excluded.

## Figures and Tables

**Table 1:** Unstandardized and standardized estimates of the direct, indirect, and total associations.

Type	Effects	B	Robust SE	95% CI for B	*β*	Z	*p*	% Mediation
Variables	PI ⇒ R	−0.671	0.051	[−0.772, −0.570]	−0.557	−13.045	0.001	
	R ⇒ PD	−0.232	0.051	[−0.332, −0.132]	−0.131	−4.546	0.001	
Direct	PI ⇒ PD	1.713	0.116	[1.485, 1.940]	0.803	14.749	0.001	91.7%
Indirect	PI ⇒ R ⇒ PD	0.156	0.035	[0.088, 0.223]	0.073	4.509	0.001	8.3%
Total	PI ⇒ PD	1.868	0.112	[1.648, 2.088]	0.876	16.638	0.001	100%

Note. R = resilience, PI = psychological inflexibility, PD = psychological distress, B = unstandardized coefficient; Robust SE = robust standard error; 95% CI for B = 95% confidence interval for the unstandardized coefficient based on robust standard errors; β = standardized coefficient; z = z-score; *p* = *p*-value.

## Data Availability

The data presented in this study are available upon request from the corresponding author due to privacy issues.
